# Are You Prepared to Save a Life? Nursing Students’ Experience in Advanced Life Support Practice

**DOI:** 10.3390/ijerph18031273

**Published:** 2021-01-31

**Authors:** Lorena Gutiérrez-Puertas, Verónica V. Márquez-Hernández, Vanesa Gutiérrez-Puertas, Mª Carmen Rodríguez-García, Alba García-Viola, Gabriel Aguilera-Manrique

**Affiliations:** 1 Department of Nursing, Physiotherapy and Medicine, Faculty of Health Sciences, University of Almeria Sacramento S/N, en La Cañada de San Urbano, 04120 Almería, Spain; lgp524@ual.es (L.G.-P.); vgp919@ual.es (V.G.-P.); mrg451@ual.es (M.C.R.-G.); albagarciaviola@hotmail.com (A.G.-V.); gaguiler@ual.es (G.A.-M.); 2 Research Group of Health Sciences, CTS-451, 04120 Almería, Spain

**Keywords:** advanced life support, cardiac arrest, hospital, nursing students, qualitative research

## Abstract

The objective of this study was to explore the experiences and perceptions of nursing students after applying advanced life support techniques on a hospitalised patient in cardiac arrest in a simulated setting. A qualitative descriptive phenomenological study was conducted. Fifty-four nursing students from the University of Almería (Spain) participated. Three main themes and six subthemes were identified, which illustrate the experiences and perceptions of nursing students about performing advanced life support. The main themes were: (1) Analysing practice as part of the learning process, with the subthemes “working in an unknown environment” and “acquiring knowledge as the key to success”; (2) Facing reality: nursing students’ perceptions of an emergency situation, with the subthemes “facing stressful elements” and “emotional impact in emergency situations”; (3) Experience as a key element to integrating advanced life support into the healthcare setting, with the subthemes “discovering and facing the experience as a team” and “linking and transferring the situation to a real clinical setting”. The nursing students reported that the process of practising for an emergency situation through simulation was a fundamental part of their training, as it allowed them to acquire skills necessary for emergency situations and improve their clinical performance in advanced life support. In addition, they considered the experience a key element in integrating advanced life support into the healthcare setting. The results of this study highlight the need to develop and implement training programs focused on clinical and teamwork skills in nursing programs.

## 1. Introduction

Students in nursing programs face a great challenge, as they must master both didactic content and clinical skills [1]. The clinical setting allows the students to practise on patients what they have learned in the nursing-practice laboratory [2]. However, nursing students have difficulties developing skills in clinical procedures during their clinical practice [3], such as performing cardiac arrest techniques. Therefore, it is necessary for university professors to provide sound training in clinical skills, based on innovative teaching methodologies, in order to guarantee patient safety and foster self-confidence in the students when carrying out clinical procedures on patients [4].

Cardiac arrest is a time-dependent, high-acuity event, which requires the coordination of various healthcare professionals at once to optimise the success of cardiopulmonary resuscitation (CPR) [5]. The organisation of the team in the hospital environment becomes a challenge due to the stress that comes from performing advanced life support (ALS) [6]. Similarly, compliance with ALS guidelines is related to a higher rate of return of spontaneous circulation, which is key to preventing patient deterioration and minimising complications [7]. Furthermore, adjusting to the recommended times, as well as determining the optimal time to monitor the rhythm, epinephrine and defibrillation are important aspects that could improve the team’s performance [5]. High-quality CPR could positively affect patient outcomes and survival rates [8,9]. Other aspects that could optimise ALS performance are team communication [10] and leadership skills [11].

Advanced life support skills, as well as teamwork, are essential to healthcare professionals, which is why ALS training and practice are needed [7]. The American Heart Association (AHA) guidelines for cardiopulmonary resuscitation (CPR) recommend that students in health professions be trained in advance in ALS techniques [12]. Specifically, ALS training continues to be a vital part of nursing education and requires an adequate intervention to guarantee that nurses feel competent and are capable of acting when faced with individuals in cardiac arrest [13]. Moreover, nursing curricula must incorporate elements such as CPR techniques into the early stages of the students’ education, with the aim of generating self-confidence and preparing the students for this important procedure in clinical practice [14], although several studies show the majority of nursing students have suitable ALS skills [15,16].

Furthermore, a high-fidelity simulation could be an efficient way to learn actively and practice the ALS guidelines in a safe and reproducible environment [17]. This type of simulation may improve knowledge, acquisition of technical and non-technical skills, and teamwork and self-confidence in performing ALS techniques [15]. Similarly, ALS may have to be performed in a variety of clinical situations. Thus, simulation would enable students to develop situational awareness, which is an important aspect of carrying out ALS guidelines in the clinical environment [18]. Previous studies have shown that simulation positively affects nursing students’ learning and competency acquisition for managing emergency situations, like ALS [19]. Furthermore, simulation based on ALS improves knowledge retention and skills in nursing students [20] and minimizes the time it takes to act during these situations [15]. However, there are no studies that explore the experience of nursing students in the application of ALS techniques, considering the stressful situation they must face while working as a team, in order to identify the factors involved in performing ALS techniques, which is the novel nature of this study.

The educational framework for this simulation workshop was based on Kolb’s Experiential Learning Theory. Following the basic concepts of this theory, the students who participated in the simulation experienced a real-life situation. Afterwards, the students reflected on their actions during the debriefing session to analyse the experience and get feedback on how they felt during the activity. Moreover, the students were encouraged to reflect on what they could have done differently and generalize those topics to other situations, which allowed an abstract conceptualization that could be extrapolated to similar situations [21].

As previously mentioned, the application of ALS techniques in the clinical setting are essential for nursing students, given that nursing professionals are the first to act in these types of situations [22]. In addition, previous experience has been shown to be crucial to the success of carrying out ALS techniques [23]. However, there are no studies that explore the experience of nursing students in the application of ALS techniques, taking into account the stressful situation they must face, while working as a team. Therefore, the objective of this study was to explore the experiences and perceptions of nursing students after applying advanced life support techniques on a hospitalised patient in cardiac arrest in a simulated setting.

## 2. Materials and Methods

### 2.1. Study Design

A descriptive phenomenological approach was used as a guide to enable a rich understanding of the participants’ life experiences. Phenomenology was created to explore the meanings of live experiences, to be directly open to phenomena and to be able to perceive “the things” themselves [24]. In Husserl’s descriptive phenomenology, in order to clarify the life-world, researchers must put aside prior knowledge and preconceptions as much as possible so that descriptions can reveal unexpected connections and meanings [25]. The study was presented in accordance with the Consolidated Criteria for Reporting Qualitative Research (COREQ) checklist [26].

### 2.2. Participants

The nursing students were from the University of Almería (Spain). The nursing students were selected through an intentional convenience sample and were not given economic compensation for their participation. The inclusion criteria established were: (a) being enrolled in the subject Basic Life Support (BLS) and ALS, and (b) having attended all the previous training sessions on BLS and ALS. The exclusion criteria included: (a) being a foreign exchange student, in order to ensure complete understanding of the experience, and (b) being a healthcare professional with experience in CPR.

### 2.3. Data Collection

Data collection took place in a laboratory in the Health Sciences Faculty. Data collection included 6 focus groups (FGs) and took place in March 2020. The focus groups were moderated by the lead researcher. The FGs allowed participants to express their perceptions and experiences in a spontaneous way and to reflect on them, generating an exchange of ideas [27]. Previously, a script with open questions was developed, based on a review of the literature (Table 1). Each FG included nine nursing students who agreed to discuss and share their simulation experiences in applying ALS to a hospitalised patient who went into cardiac arrest.

**Figure 1:**
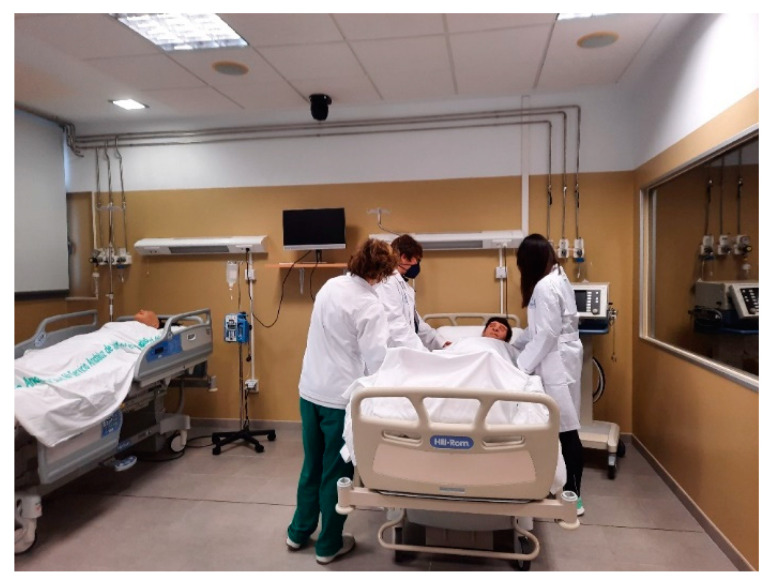
The simulated scenario.

The simulated scenario (Figure 1) was developed according to the recommendations of the International Nursing Association for Clinical Simulation and Learning (INACSL) standards of best practice in simulation [28]. Before participating in the simulation, the participants attended six informative sessions about ALS procedures and received training on the guidelines to follow when assisting a patient who goes into cardiac arrest in a hospital setting. The pre-briefing included an orientation on the expectations of the students, and they were informed that the aim of the simulation was for training purposes, although they would be evaluated as well, as the evaluation formed part of the training process. Before the students began the simulation, a group discussion was held on ALS intervention protocols. The lead researcher briefed the students on the patient’s current hospital situation. The scenario focused on ALS skill development. Actors assumed the role of the patient’s voice, having been instructed in a standardised orientation session on how to perform their roles. The assignment of teams, as well as the roles of nursing students within the teams, was done by the professors of the subject. The scenario began with a nursing student, who was in a hospital room with a patient. The patient reported feeling poorly, and after a few seconds, the patient lost consciousness and became unresponsive. The nursing student that was in the room had to call for help, and then two other nursing students would join the first student. One of the three students took on the role of leader before beginning their simulation. The observers watched the scene from a separate room through a video screen. The observers were registered nurses who had experience in CPR as well as basic life support- advanced life support (BLS-ACLS) training. The role of the observers was to document the interventions completed by the nurses and identify elements of teamwork. Subsequently, once all of the simulations were carried out, the lead researcher held a debriefing session to analyse the experience and get feedback on how the nursing students felt throughout the activity.

The FGs were monitored by the lead researcher. The duration of the FGs varied from 20 to 40 min. Sociodemographic data was collected from all participants before beginning the FGs. The responses of participants in FGs were audio-recorded and transcribed [27]. Data collection through the FGs ceased when data saturation was reached.

### 2.4. Data Analysis

For data analysis, the transcriptions were included in a hermeneutic unit and analysed using the Atlas-ti 8.0. software. To ensure the reliability and validity of the results, the researchers followed the Colaizzi [29] methods of descriptive phenomenological data analysis: (1) Completed transcript of the interview and understood the participants´ lived experiences; (2) Significant sentences were scrutinized to meaningful statements; (3) Meaningful statements were extracted to meaningful units; (4) Meaningful units were classified into subthemes and themes; (5) Themes and subthemes were integrated into a comprehensive description of the participants´ lived experiences; (6) The basic structure of the participants´ lived experiences was described; (7) Two interviewees analysed the findings for verification of the accuracy of the transcripts and resemblance of their experiences.

### 2.5. Ethical Considerations

The study was approved by the Ethics and Research Committee at the university where it was carried out (EFM-62/20). All participants were previously informed about the objective of the research as well as the voluntary nature of their participation. The participants signed an informed consent form prior to starting their interviews. In addition, participants were asked for permission to record their conversations and given access to the results of the study. To ensure anonymity and confidentiality of the data, all interviews received codes. The guidelines of the Declaration of Helsinki were followed at all times.

### 2.6. Rigour

Finally, reliability and rigour were established for the qualitative data. With the aim of increasing reliability, data triangulation was performed by three of the researchers (L.G.P., V.G.P., and V.M.H.), which included analysing the data separately and discussing the differences until a consensus was reached, in order to select themes and subthemes. A separate researcher (G.A.M.) read the transcriptions of the FGs to confirm their concurrence with the findings obtained (themes and subthemes), verifying that all of the participants’ perspectives were considered. The recordings, data analysis and interviews were saved to ensure reliability. The participants verified the transcriptions and data analysis, ensuring their confirmability.

## 3. Results

The total study sample consisted of 54 nursing students, distributed into 6 FGs. Of the participants, 75.9% (*n* = 41) were female and 24.1% (*n* = 13) were male. The average age was 20.63 years old (SD = 4.42; range = 18–46). The sociodemographic characteristics of participants can be seen in Table 2.

The three main themes, subthemes and units of meaning that emerged in the analysis are presented in Table 3.

### 3.1. Analysing Practice as a Part of the Training Process

One of the main themes that emerged from the interviews was the importance of simulation in nursing training. This meaning is based on the evaluation that nursing students make of their performance, in a setting which is full of uncertainty for them, as part of the learning process. The participants reported that the simulation allowed them to acquire key skills for successful ALS. This theme contains two subthemes, discussed below.

#### 3.1.1. Working in an Unknown Environment

The nursing students identified their work environment as one of the most essential elements to the successful performance of ALS guidelines. At the beginning of the simulation, participants reported feeling out of place when facing an unknown environment, which caused difficulties in initiating ALS manoeuvres. Similarly, participants highlighted the realism of the environment in which the simulation occurred. This realism helped the students to fully immerse themselves in the situation, and to feel like independent nursing professionals with self-reliance and responsibility over the procedures that they carried out, regardless of being evaluated.

“*I felt a bit lost at the beginning and it was like...oh dear, what do I do now? I didn’t know where to start, I felt out of place and in a crisis situation, where you have to act quickly…*”

(G3-P2, female, 19 years old)

“*It was all really realistic, and that helps, because you take on the role more, you really get into it, you feel as if you were actually the patient’s nurse, and it shows you how you would react… I forgot that there were people evaluating us, I just thought about doing my best, because the patient’s life depended on us.*”

(G1-P5, female, 20 years old)

Some participants commented that feeling observed did not negatively interfere with them carrying out ALS, but rather, it increased their expectations for themselves, as they felt that their ability to save someone’s life was being evaluated.

“*You know they’re watching you, but it’s not distracting, although I did feel questioned. That [feeling observed] makes you take it more seriously.*”

(G3-P6, female, 19 years old)

#### 3.1.2. Acquiring Knowledge as the Key to Success

One of the aspects which the participants indicated could be improved in the training process was the integration of theoretical concepts into practice. The participants noted that, despite having received theoretical training in ALS, when it came to the real situation, they had trouble applying that knowledge.

“*Theory is one thing, and how you apply it is another. When it comes time to really do it, you say, what do I have to do? And that... how do I do it again?... With everything they’ve taught us about the rhythm, we still got it wrong when identifying it...*”

(G2-P8, female, 25 years old)

Some participants reported that the simulation allowed them to incorporate various techniques into one situation. This led students to comprehend the complexity of the clinical work that nursing professionals have to deal with when addressing emergency situations.

“*In our practical classes, you focus on a certain intervention, for example, practice about the Ambu^®^ is only about the Ambu^®^. Here [in the simulation] you focus on several different things… you see the complexity of the work [of nursing professionals] and you realise all that it entails.*”

(G4-P7, male, 23 years old)

The debriefing allowed the students to reflect on their performance and detect any aspects they needed to improve for similar future situations. This led students to recognise simulation as the learning methodology that could be most beneficial to completing their training in ALS.

“*The fact that they [the professors] tell you the mistakes you have made so you know if you’ve performed well on the things you studied… that way you can learn from your mistakes... This [the debriefing] helps you see what you’ve done wrong so you can correct those mistakes and learn more than you would otherwise.*”

(G2-P9, female, 20 years old)

### 3.2. Facing Reality: Nursing Students’ Perceptions in an Emergency Situation

This category explored the stressful factors perceived by the students, as well as the emotional impact that they experienced when facing an emergency situation. The students identified several stressful elements that interfered with performing ALS, which made it more difficult to achieve the desired outcome of saving the patient’s life.

#### 3.2.1. Facing Stressful Elements

The participants noted that they had to face several stressful elements during the simulation. The students had preconceived ideas about the complexity of the situation, as well as about the work that nursing professionals do. When faced with the complexity of the situation, the students reported having difficulty concentrating and an inability to carry out interventions in a simultaneous way.

“*You imagine that it’s going to be easier, you don’t think that one nurse is capable of doing everything that has to be done. You have to be aware of so many things, you have a lot weighing on you… you feel a bit lost, overwhelmed, incapable, and that leads to mistakes...nothing turned out as I hoped it would, I couldn’t concentrate...my heart was pounding.*”

(G3-P7, female, 21 years old)

In addition, the fact that students had to perform the steps of ALS rapidly, due to the importance of time in the success rate of CPR, made them work quickly, yet not as efficiently.

“*The person is dying, so ti5,35 cmme is crucial in saving them...you feel like you can’t go any faster...so you start rushing, miss steps, you get more and more nervous...you see that time is running out...that they’re going to die.*”

(G6-P9, male, 20 years old)

#### 3.2.2. Emotional Impact in Emergency Situations

When faced with the emergency situation, participants experienced an emotional impact that manifested itself through various feelings that arose throughout the practice. These feelings stemmed from the fact that they wanted to save the patient’s life, but their inexperience in making decisions autonomously hindered their performance in the interventions. Some participants noted feeling a fear of failure, which led them to want to release their feelings of guilt. Others spoke about the nervousness that they felt, which gave them insecurity upon taking action, which increased their stress levels, as they felt they were not helping the patient.

“*Fear just took over… we were just looking around guiltily [at each other] … we were failing.”*

(G3-P2, female, 19 years old)

Another feeling expressed by the students was distress, caused by the uncertainty of the situation and by perceiving that the results were beyond their control, increasing their anxiety. Similarly, their inability to save the patient’s life was taken as a personal matter, causing them to feel disappointed with themselves, which manifested as a sense of frustration.

“*We felt like crying… I feel frustrated and disappointed in myself… I just kept making mistakes… I couldn’t save his life… I just wasn’t able to.*”

(G6-P2, female, 19 years old)

### 3.3. Experience as a Key Element to Integrating ALS in Care Settings

This theme analysed the students’ perception of the teamwork needed to perform the ALS steps, as well as the transfer of the simulation experience to a clinical setting. It includes the subthemes “discovering and facing the experience as a team” and “linking and transferring the situation to a real clinical setting”.

#### 3.3.1. Discovering and Facing the Experience as a Team

The experience of working as a team was regarded as revealing for the participants. This experience allowed a greater understanding of the importance of teamwork in providing adequate care and achieving desired patient outcomes. Students recognised that effective communication was key to having positive interactions and improving decision-making during the process of ALS. A lack of communication led students to make decisions independently, generating conflicts between them, which decreased their efficiency.

“*[The members of the team] we communicated well, we told each other what we were doing as we did it and any changes that occurred in the patient... agreeing on the different actions to take...we were coordinated...it went well for us.*”

(G6-P3, male, 22 years old)

“*If you don’t communicate, it’s chaos, everyone does their own thing, which is what happened to us... we were both preparing the same material... we were wasting our time... And she [my classmate] got mad at me.*”

(G1-P8, female, 32 years old)

The participants considered the leader to be the person responsible for directing, organising, prioritising and assigning actions to every member of the team, facilitating the cohesion of the team, in order to guarantee the success of their performance and to save the patient’s life. Some participants recognised their inability to be a leader.

“*I believe it is important that one person lead because that way we followed a specific order, we prioritised…we worked in a coordinated way and…everything was focused on saving the patient.*”

(G6-P2, female, 19 years old)

“*I’m not used to giving my classmates orders… I’m not good at it…Not everybody can be a leader, you have to have certain qualities…know how to communicate…have experience, I mean, you have to have done it more.*”

(G5-P8, female, 20 years old)

Rapport was another central aspect to the success of working as a team. Some participants commented that working as a team minimised the pressure of the situation, as they perceived support from their peers and felt that responsibility was shared. Other participants stressed the importance of knowing your team members in assigning each person the most appropriate role.

“*It doesn’t just depend on you, the responsibility is everyone’s [the team], so you feel supported...if you miss something, there are other people who can do it… you feel less pressure.*”

(G6-P6, female, 46 years old)

“*You know them [your colleagues], you know what each person does best.*”

(G2-P7, female, 21 years old)

#### 3.3.2. Linking and Transferring the Situation to a Real Clinical Setting

The simulation formed part of their prior experience and allowed students to become more aware of emergency situations, which made them feel more prepared to attend to a patient who goes into cardiorespiratory arrest. This preparation was based on improving clinical skills and learning how to work as a team. The participants felt that the experience positively influenced their clinical performance, as it allowed them to recall their previous actions and be more efficient in following ALS guidelines in the hospital environment, improving the clinical safety of the patient. In addition, the participants also pointed out that they would benefit from additional simulation experiences in facing complicated clinical scenarios, which they sometimes are not exposed to during their training, in order to improve their professional work in clinical settings.

“*This [the simulation] has helped us greatly improve our skills, the teamwork makes you feel more…prepared for your practice or…for your future job…I think that if we did it all again right now, we would do it…a lot better, faster…we’ve gained experience.*”

(G5-P7, female, 19 years old)

Some participants pointed out that the simulation increased their self-confidence in coping with emergency situations in a hospital setting. However, another participant said that the experience actually decreased their self-confidence due to their inability to act in a stressful situation. The psychological impact of the experience led the student to question if nursing was the right profession for her.

“*… because, until you find yourself in the situation, you don’t trust yourself, you’re not aware of how much you can really do.*”

(G3-P5, male, 20 years old)

“*I realised that the situation was just too much for me… I find it very difficult to adapt to stressful situations… I didn’t feel comfortable…I don’t know if this is my thing [referring to the nursing profession].*”

(G6-P7, female, 19 years old)

## 4. Discussion

The objective of this study was to explore the experiences and perceptions of nursing students after applying advanced life support techniques on a hospitalised patient in cardiac arrest in a simulated setting. Firstly, the nursing students highlighted the realism of the situation, which led them to become disorientated and stood in the way of starting ALS steps. Likewise, some nurses reported having difficulty starting CPR in an unknown setting, which could influence patient outcomes [30]. Conversely, the students commented that the fact that they were being observed did not negatively interfere with their ALS performance; rather, that it increased their personal motivation when they saw that their skills were being questioned. However, no studies were found that address how feeling observed might influence nursing students’ performance. Students also indicated that they forgot that they were being evaluated during the simulation exercise, and they became fully immersed in the role of the nurse in an emergency situation. In addition, simulation allows students to put themselves into the position of a nursing professional, minimising the anxiety they feel towards being evaluated, by focusing solely on their professional performance [31].

Regarding the training process, the students indicated that simulation allowed them to link theory with practice and assimilate various clinical procedures. Simulation translates nursing students’ knowledge into better performance of clinical skills [15]. Additionally, the nursing students considered the debriefing essential to encouraging significant learning, as it helped them identify aspects that they need to improve in their performance of ALS techniques. The implementation of the debriefing after performing CPR gives students the opportunity to exchange ideas to improve their technique or teamwork skills, with the aim of optimising their performance and improving patient outcomes [31].

Nevertheless, participants identified multiple stressors that influenced their ability to perform according to ALS guidelines. This stress could be due to a lack of awareness of the emergency situation, as well as having to respond immediately [32]. Stress can affect the clinical performance of nursing students, so students’ exposure to similar stressful experiences during the training process is essential in order to achieve optimal performance [33]. In addition, the experience had a negative emotional impact on students. This aspect has been fairly unexplored, as only one study has analysed the emotional impact of simulating an emergency situation on medical students [34], and its results coincide with the data reported by the participants in this study. Similarly, simulation programs often do not address potential emotional issues that participants face when dealing with stressful situations [35]. Nursing students should therefore be trained in emotional management to address stressors during high-acuity events.

Nursing students stressed the importance of teamwork in providing adequate care and achieving desired patient outcomes. Students also noticed shortcomings in interprofessional communication skills that negatively interfered with the team’s performance. Verbal and nonverbal communication has been shown to be vital to team coordination when performing ALS manoeuvres, reducing action time by improving teamwork skills [36]. This improvement in action time minimises the risk of neurological damage [9] and increases the patient’s chances of survival [8]. Furthermore, many students reported not feeling capable of being a leader. Previous studies showed that simulation enables leadership training by improving cooperative skills during CPR [37,38]. It is therefore imperative that nursing students apply effective communication techniques, such as the implementation of closed-loop communication. This technique has been shown to improve communication between team members in emergency situations [33].

The students reported that the experience could be applied to a hospital setting, as they felt more capable of following the ALS guidelines due to an increase in their confidence and clinical skills. Additionally, the closer the situation and the simulation setting are to reality, the more likely the learned behaviour is to be transferred to the clinical environment [18]. In addition, students indicated that the experience would allow them to perform better and improve the health care they provide. In fact, when simulation experiences are comparable to real-life clinical practice situations, students can accurately recall the actions they have taken, which has a positive influence on patient safety [39]. Increased exposure to simulated scenarios could improve CPR performance [17]. In addition, students do not have the opportunity to address numerous clinical situations during their clinical practice, facing them for the first time as nursing professionals. Because of this, simulation allows nursing students to safely cope with clinical situations in which they may find themselves immersed, which provides them with prior training for later use in the clinical environment [15].

However, some students went so far as to question if they could handle being nursing professionals after facing an emergency situation. This perception could be due to the stress caused by the situation and a lack of the skills required to successfully carry out the procedure [34]. Thus, it would be necessary to address the psychological impact that facing stressful clinical situations can have and how this can interfere with the performance of their clinical work as a future nursing professional, which has not been addressed in previous studies. Furthermore, there are no studies that explore the experience of nursing students in the application of ALS techniques, considering the stressful situation they must face while working as a team, in order to identify the factors involved in performing ALS techniques and develop strategies that promote student success when performing ALS techniques in the clinical setting. The results of this study highlight the need to develop and implement training programs focused on clinical and teamwork skills in nursing programs, in order to train future nursing professionals in addressing emergencies, as well as the need to develop measures to evaluate and minimize the psychological impact that facing stressful situations can have on the students in their future work as a nursing professional.

### Limitations

The results of this study should be considered in the light of some limitations, such as sample size and participant selection, as the sample was drawn from a single institution. On the other hand, the design of the study does not aim to generalise the results, but to explore the experiences and perceptions of nursing students in the development of ALS in the hospital environment, which are relatively unknown. Future research could involve a broader representation of participants from different nursing faculties in order to gain greater diversity of experience and greater confidence in the transferability of the findings.

## 5. Conclusions

Nursing students identify the simulation of an emergency situation as a critical part of their learning process. In addition, they consider experience a key element in integrating ALS into the care environment. Training future nursing professionals and providing them with the skills they need to face emergency situations would aid in their familiarisation with the hospital setting and improve their clinical performance of advanced life support manoeuvres.

## Figures and Tables

**Table 1:** Guide questions used for the focus groups.

Guide Questions
Could you tell us about your first experience of advanced life support in the hospital environment? How do you think this experience has influenced in your clinical training? Could you tell me if you have detected elements that interfere with the development of advanced life support procedures? Which have they been? Why? How have you felt acting in this emergency situation? Why? Tell me about your experience working as a team What teamwork factors have you detected that influences the adequate development of advanced life support? How do you think this experience could help you develop advanced life support in the clinical practices? What are your general impressions of the experience? Is there anything else you would like to add about this theme?

**Table 2:** Socio-demographic data of the participants (*n* = 54).

Variable	Total (*n* = 54)	
	*n*	%
*Sex* Male Female	13 41	24.1 75.9
Age	20.63 *	4.42 **
*Previous Training Basic PCR* Yes No	8 46	14.8 85.2

* Mean ** Standard Deviation.

**Table 3:** Units of meaning, subthemes and main themes of the analysis.

Units of Meaning	Subthemes	Main Themes
Out of place, contextualization of the realistic environment, abstraction of the evaluation, feeling observed.	Working in an unknown environment.	Theme 1. Analysing practice as a part of the training process.
Theory-practice gap, integration of procedures, meaningful learning, satisfactory experience.	Acquiring knowledge as the key to success.	
Preconceived ideas, task simultaneity, psychological pressure, time.	Facing stressful elements.	Theme 2. Facing reality: nursing students’ perceptions in an emergency situation.
Fear, nervousness, overwhelmed, frustration.	Emotional impact in emergency situations.	
Communication, leadership, work a team.	Discovering and facing the experience as a team.	Theme 3. Experience as a key element to integrating ALS in care settings.
Clinical training, clinical performance, self-confidence, improved healthcare.	Linking and transferring the situation to a real clinical setting.	

## Data Availability

Data available on request due to restrictions.
